# HiC-Hiker: a probabilistic model to determine contig orientation in chromosome-length scaffolds with Hi-C

**DOI:** 10.1093/bioinformatics/btaa288

**Published:** 2020-05-05

**Authors:** Ryo Nakabayashi, Shinichi Morishita

**Affiliations:** Department of Computational Biology and Medical Sciences, Graduate School of Frontier Sciences, The University of Tokyo, Chiba 277-8562, Japan

## Abstract

**Motivation:**

*De novo* assembly of reference-quality genomes used to require enormously laborious tasks. In particular, it is extremely time-consuming to build genome markers for ordering assembled contigs along chromosomes; thus, they are only available for well-established model organisms. To resolve this issue, recent studies demonstrated that Hi-C could be a powerful and cost-effective means to output chromosome-length scaffolds for non-model species with no genome marker resources, because the Hi-C contact frequency between a pair of two loci can be a good estimator of their genomic distance, even if there is a large gap between them. Indeed, state-of-the-art methods such as 3D-DNA are now widely used for locating contigs in chromosomes. However, it remains challenging to reduce errors in contig orientation because shorter contigs have fewer contacts with their neighboring contigs. These orientation errors lower the accuracy of gene prediction, read alignment, and synteny block estimation in comparative genomics.

**Results:**

To reduce these contig orientation errors, we propose a new algorithm, named HiC-Hiker, which has a firm grounding in probabilistic theory, rigorously models Hi-C contacts across contigs, and effectively infers the most probable orientations via the Viterbi algorithm. We compared HiC-Hiker and 3D-DNA using human and worm genome contigs generated from short reads, evaluated their performances, and observed a remarkable reduction in the contig orientation error rate from 4.3% (3D-DNA) to 1.7% (HiC-Hiker). Our algorithm can consider long-range information between distal contigs and precisely estimates Hi-C read contact probabilities among contigs, which may also be useful for determining the ordering of contigs.

**Availability and implementation:**

HiC-Hiker is freely available at: https://github.com/ryought/hic_hiker.

## 1 Introduction

High-quality reference genome sequences have been essential in analyses of organisms over the last two decades; however, due to the prevalence of repetitive elements in genomes, it is still challenging to rebuild the original genomes from reads of short DNA fragments. Specifically, if we use the widely accepted whole-genome shotgun sequencing strategy (Weber and Myers, 1997) with short reads, we can collect short reads and assemble overlapping reads into contiguous sequences, named contigs, using genome assemblers (Butler *et al.*, 2008; Gnerre *et al.*, 2011; Luo *et al.*, 2012; Zerbino and Birney, 2008). The overlapping step, however, is likely to stop extending contigs when it hits abundant repetitive regions that make the extending step ambiguous. Thus, contigs from short reads are fairly short, i.e. typically <100 kb in size, such that an alternative approach is needed for ordering contigs along chromosomes. To this end, methods for linking neighboring contigs such as bacterial artificial chromosome (or fosmid) end-sequence pairs (Edwards *et al.*, 1990; Venter *et al.*, 2001), long reads (Eid *et al.*, 2009; Loose *et al.*, 2016) and 10× read clouds (Weisenfeld *et al.*, 2017) have been used to output scaffolds of contigs, although these methods fail to produce the chromosome-length scaffolds of large mammalian genomes. To obtain chromosome-length genome sequences, many projects, including the human and worm genome sequencing projects (Lander *et al.*, 2001; The *C. elegans* Sequencing Consortium, 1998), have attempted to generate genome markers for anchoring scaffolds on chromosomes; however, this is a time-consuming and expensive task.

To overcome these issues in genome assembly, many more links between proximal contigs are needed. A recent promising approach exploits the fact that long DNA molecules are packed in a small nucleus, where a pair of proximal loci are more likely to interact than a distal pair. This tendency can be quantified approximately by the Hi-C method, which allows us to measure the contact frequency between pairs of loci in a genome-wide manner (Lieberman-Aiden *et al.*, 2009). Indeed, the contact frequency between a pair of loci, as observed by Hi-C, is proven to be correlated with the one-dimensional distance between the pair (Lieberman-Aiden *et al.*, 2009), allowing us to use Hi-C data as linking information. This approach can connect distal positions at distances of over 1 million base pairs, including regions of low recombination frequency such as those surrounding meta-centromeres (Dudchenko *et al.*, 2017). The basic concept was proposed in 2013 (Burton *et al.*, 2013) and has been improved by subsequent studies (Dudchenko *et al.*, 2017; Ghurye *et al.*, 2019; Putnam *et al.*, 2016; Zhang *et al.*, 2018, 2019). The software program 3D-DNA has shown high performance in producing high-quality chromosome-length scaffolds for human and *Aedes aegypti* genomes (Dudchenko *et al.*, 2017). The 3D-DNA software is used by the *DNA-ZOO* project, which is aiming to produce chromosome-length genomes of many non-model organisms at under $1000 per each organism using short-read sequencing data and only 7× coverage Hi-C data (Dudchenko *et al.*, 2018).

The 3D-DNA package performs best in terms of accuracy, particularly for contigs assembled from short reads and without prior knowledge of the number of chromosomes; however, it can output scaffolds containing inverted, translocated or misoriented short contigs (Dudchenko *et al.*, 2017, 2018). A larger error is easier to identify by manual inspection than a smaller one, if the contact frequency matrix of the focal region is visualized to facilitate manual revision. To assist such investigation, 3D-DNA is equipped with a graphical user interface (GUI) to help users interactively identify large errors in an assembly and fix them manually (Dudchenko *et al.*, 2018). With this GUI, the user can correct many of the large errors in genome assemblies, although it remains infeasible to manually repair a number of misoriented short contigs, which often hinder gene prediction and disturb comparative genomic analysis.

**Fig. 1.**
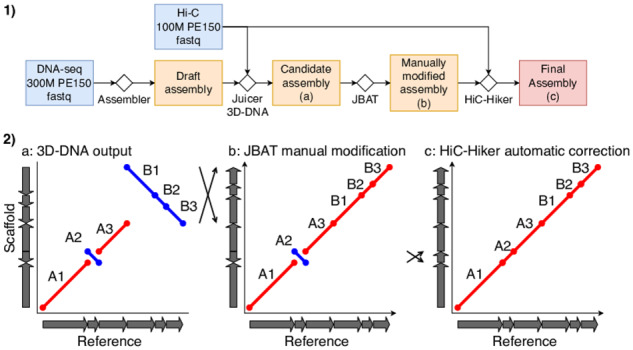
(**1**) A typical workflow of *de novo* assembly using 3D-DNA and HiC-Hiker. HiC-Hiker is used as a post-processing step of the current 3D-DNA pipeline to correct local orientation errors. (**2**) (**a**), (**b**) and (**c**) show schematic dot plots (reference on the x-axis; three scaffolds on the y-axis) in the assembling procedure of an example scaffold composed of six mock contigs. The red and blue lines in the dot plots represent forward- and reverse-complement alignments of scaffolds, respectively. The arrays of arrows on both axes represent the ordering and orientation of contigs in the reference and scaffolds. The leftmost dot plot (a) illustrates 3D-DNA chromosome-length scaffolds with two major errors; the top-right large inversion that involves contigs B1–B3 and the misoriented short contig labeled A2. The former large inversion error can be corrected using Juicebox Assembly Tools (JBAT), since such large errors are typically apparent in plots of the Hi-C contact frequency matrix (b). In contrast, the latter, minor error is often difficult to detect in the contact matrix, so we propose the use of HiC-Hiker to fix the small misorientations; (c) shows revised scaffolds

Thus, we developed a new method that is capable of correcting misoriented contigs automatically to improve the output of 3D-DNA (Fig. 1). The HiC-Hiker package can apply file formats used by the 3D-DNA and Juicer packages (e.g. assembly files for scaffold layout and merged_nodups.txt files for Hi-C read alignment), as well as generic formats (e.g. AGP files for scaffold layout and SAM or BAM files for Hi-C read alignment), which are used by several Hi-C-based scaffolding tools. Our probabilistic model of a Hi-C contact probability considers Hi-C contacts not only between two adjacent contigs, but also those within *k* neighboring contigs. Because the contact probability distribution is dependent on each sample (Lieberman-Aiden *et al.*, 2009), it is crucial to obtain a better, empirical approximation of the distribution from a given Hi-C dataset.

To calculate an optimal series of oriented contigs with the globally maximum probability, we here revisit and revise the idea of dynamic programing algorithm used by the Chicago library (Putnam *et al.*, 2016), provide a rigorous mathematical model and extend the algorithm to the processing of contigs of uneven length. We adapt the model to be applicable to scaffolds produced by 3D-DNA and Hi-C data, and achieve better performances compared to previous methods for real human and worm genome datasets.

## 2 Materials and methods

### 2.1 Formalization of the contig orientation optimization problem

We first present a formal definition of the scaffolding problem. A *scaffold* is an ordering of contigs, while a *super-scaffold* is a scaffold whose contigs are located in its corresponding chromosome; it is called a *chromosome-level* scaffold if it covers most parts of the chromosome. We here divide the contig scaffolding process into two steps; namely, approximate layout of contigs along chromosomes and precise refinement of contig orientations. We assume a situation where the first step has been done and the relative positions of contigs on the super-scaffold are determined by state-of-the-art software programs, such as 3D-DNA. Thus, we focus on the second step of estimating the optimal orientation of each contig. This problem setting is realistic because 3D-DNA has proven capable of ordering contigs with a high degree of accuracy (Dudchenko *et al.*, 2018). The one remaining major issue is reduction of contig orientation errors.

**Fig. 2.**
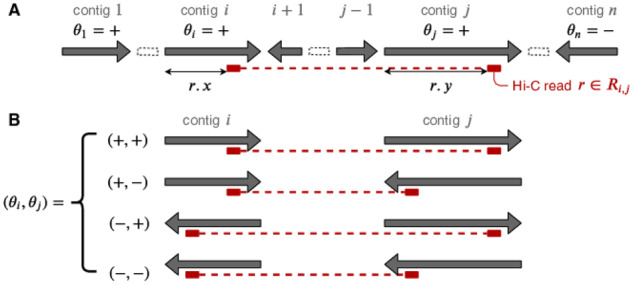
(**a**) A schematic representation of a scaffold and a Hi-C contact in our formalization. (**b**) There are four cases orienting two contigs, i.e. the *i*th and *j*th contigs; in each case, the distance between the contact points of $r\in R_{i,j}$ is fixed depending on $\theta_{i},\theta_{j}$, as illustrated by the lengths of the red dotted lines

Suppose that we have a chromosome-length super-scaffold in which *n* contigs are ordered. For i=1,…,n, the *i*th contig is a nucleotide sequence of length *L_i_* bp, and its orientation in the super-scaffold is denoted as $\theta_{i}\in \{+,-\}$, where $\theta_{i}=-$ implies that the contig is reverse complemented in the scaffold (see Fig. 2). Let $R_{i,j}$ be a set of Hi-C contacts that connect the *i*th and *j*th contigs. Each contact $r\in R_{i,j}$ is a pair of positions on the *i*th and *j*th contigs, $r\cdot x\in [0,L_{i})$ and $r\cdot y\in [0,L_{j})$. The entire set of Hi-C contacts between different contigs is denoted as the disjoint union $R=\underset{1\leq i<j\leq n}{⊔}R_{i,j}$. Using these notations, we aimed to estimate the best possible contig orientations $\Theta =\{\theta_{i}|i=1,\ldots ,n\}$ from all Hi-C contacts, *R*.

### 2.2 Probability of each Hi-C contact read

Next we characterize the contig orientation inference problem from a probabilistic viewpoint. A Hi-C contact frequency is known to be largely correlated with the one-dimensional base-pair distance (Lieberman-Aiden *et al.*, 2009); therefore, let *p*(*d*) be the probability that we observe a contact between two loci separated by the base-pair length *d*. There are rare exceptional situations where the contact frequency between two locations at distance *d* does not follow *p*(*d*) due to the absence of contacts in high-GC content regions, low sequence mappability in repetitive regions or inherent three-dimensional structures, such as large recurrent CCCTC-binding factor (CTCF) loops.

Despite these relatively rare exceptions, we are able to estimate the probability distribution *p*(*d*) reliably from real Hi-C contacts, although we must take into account the fact that *p*(*d*) can often differ among samples from different species, cell types and/or cell cycles. We must predict an accurate probability distribution for each sample *de novo*. To this end, we can align pairs of reads to a contig and obtain a profile of *p*(*d*) experimentally. Since longer contigs are more informative than shorter contigs, we use the longest contig to generate a contact frequency distribution between two loci at distance *d*, and to estimate *p*(*d*) using kernel density estimation. *p*(*d*) monotonically decreases in terms of *d*, and for a larger value of *d*, the estimate based on real data is likely to be unreliable simply because the number of contacts at distance *d* can be zero, or very small. Thus, with a proper threshold *K*, we set *p*(*d*) to a constant *p*(*K*) such that we observe random contacts when *d *>* K*. The choice of threshold *K* depends on the size of a given Hi-C dataset. We then examined the effect of the *K* value on output accuracy; the third quantile in the histogram of Hi-C contact separation distances provided nearly optimal accuracy (see Section 3). Thus, using the HiC-Hiker package, *K* is determined automatically from the third quantile, if not specified otherwise.

Next, we calculate the probability of each Hi-C contact between the *i*th and *j*th contigs given their orientations θ_*i*_ and θ_*j*_. Because it is difficult to predict the size of the gap between adjacent contigs, we here simply assume that there is no gap between them. Using the Hi-C contact distribution *p*(*d*) created accordingly, we can derive the probability of a Hi-C contact between the *i*th and *j*th contigs, $r\in R_{i,j}$, for each contig orientation denoted by $P(r|\theta_{i},\theta_{j})$ (see Fig. 2):

(1) $$\begin{array}{c} P(r|\theta_{i},\theta_{j})\\ =\{\begin{array}{cc} \frac{1}{Z_{i,j}}p(r\cdot x+D+r\cdot y) & \left(\theta_{i}=-,\theta_{j}=+\right)\\ \frac{1}{Z_{i,j}}p(r\cdot x+D+L_{j}-r\cdot y) & \left(\theta_{i}=-,\theta_{j}=-\right)\\ \frac{1}{Z_{i,j}}p(L_{i}-r\cdot x+D+r\cdot y) & \left(\theta_{i}=+,\theta_{j}=+\right)\\ \frac{1}{Z_{i,j}}p(L_{i}-r\cdot x+D+L_{j}-r\cdot y) & \left(\theta_{i}=+,\theta_{j}=-\right) \end{array} \end{array}$$

where $D=\sum_{i<s<j}L_{s}$ is the distance between the *i*th and *j*th contigs. $Z_{i,j}$ is a normalization factor satisfying the definition of the probability density function. Note that the normalization factors are identical among the above four cases:

$$Z_{i,j}=\{\begin{array}{cc} \int_{x=0}^{L_{i}}\int_{y=0}^{L_{j}}p(x+D+y)\mathrm{dxdy} & \left(\theta_{i}=-,\theta_{j}=+\right)\\ \int_{x=0}^{L_{i}}\int_{y\prime =0}^{L_{j}}p(x+D+L_{j}-y\prime )\mathrm{dxdy}\prime & \left(\theta_{i}=-,\theta_{j}=-\right)\\ \int_{x\prime =0}^{L_{i}}\int_{y=0}^{L_{j}}p(L_{i}-x^{\prime }+D+y)dx\prime dy & \left(\theta_{i}=+,\theta_{j}=+\right)\\ \int_{x\prime =0}^{L_{i}}\int_{y\prime =0}^{L_{j}}p(L_{i}-x\prime +D+L_{j}-y\prime )dx\prime dy\prime & \left(\theta_{i}=+,\theta_{j}=-\right) \end{array}$$

where $x\prime =L_{i}-x$ and $y\prime =L_{j}-y$.

Finally, because each contact $r\in R_{i,j}$ is collected independently, the probability of a set of Hi-C contacts given the contig orientation θ_*i*_ and θ_*j*_ can be written as

$$P(R_{i,j}|\theta_{i},\theta_{j})=\prod_{r\in R_{i,j}}P(r|\theta_{i},\theta_{j})$$

Notably, because the probability of $r\in R_{i,j}$ is independent of contig orientations other than $\theta_{i},\theta_{j}$, we have

$$P(R_{i,j}|\Theta )=P(R_{i,j}|\theta_{i},\theta_{j}),$$

where $\Theta =\{\theta_{i}|i=1,\ldots ,n\}$. The probability of all Hi-C contacts between contigs in a dataset is:

$$P(R|\Theta )=\prod_{1\leq i<j\leq n}P(R_{i,j}|\Theta ),$$

where $R=\underset{1\leq i<j\leq n}{⊔}R_{i,j}$. Thus, our goal is to find an optimal instance of Θ that maximizes P(R|Θ):

$$\arg\underset{\Theta }{\max}P(R|\Theta )$$

To design a hidden Markov model (HMM) algorithm for solving this problem, we here use Bayes’ theorem to derive:

P(R,Θ)=P(R|Θ)P(Θ),

where P(Θ) is $P(\theta_{1})\cdots P(\theta_{n})$. Because the orientations of different contigs, θ_*i*_, are independent of each other and selected at random, we have $P(\theta_{i})=\frac{1}{2}$ for each $\theta_{i}\in \{+,-\}$, and we can treat P(Θ) as a constant, ${\left(\frac{1}{2}\right)}^{n}$. Thus, resolving $\arg\max_{\Theta }P(R|\Theta )$ also solves $\arg\max_{\Theta }P(R,\Theta )$. In the following, we propose an HMM algorithm for solving:

(2) $$\begin{array}{c} \arg\underset{\Theta }{\max}P(R|\Theta )=\arg\underset{\Theta }{\max}P(R,\Theta )\\ =\arg\underset{\Theta }{\max}\prod_{1\leq i<j\leq n}P(R_{i,j}|\Theta )P(\Theta ) \end{array}$$

### 2.3 Optimization of contig orientations using HMM

Hi-C contacts between more distal contigs are likely to be less informative for estimating contig orientations, because the number of contacts is small, and the distances between the contacts become independent of their orientations, which contrasts with the situation when handling proximal contigs. Our probabilistic model takes this property into account naturally; that is, since *p*(*d*) is constant for *d *>* K* if all contacts between the *i*th and *j*th contigs are at distances greater than *K*, they have the same probability regardless of their orientation. In other words, $P(R_{i,j}|\theta_{i},\theta_{j})$ is identical for all $\theta_{i},\theta_{j}\in \{+,-\}$ if $\sum_{i<s<j}L_{s}\geq K$.

For better readability, we here assume a simple condition whereby all contigs are of the same size (*L_s_* = *L* for all *s*), although this assumption is not essential and will be removed later. In this case, $\sum_{i<s<j}L_{s}\geq K$ can be simplified to |i−j|≥(K/L)+1 even in the absence of orientation information. Thus, we only need to consider contacts between *k* neighboring contigs for a given non-negative integer k=(K/L)+1; namely, a contact between the *i*th and *j*th contigs is used if |i−j|<k.

Given these conditions, we can transform the right side of Equation (2) into:

(3) $$=\arg\underset{\Theta }{\max}\prod_{\begin{array}{c} 1\leq i<j\leq n,\\ |i-j|<k \end{array}}P(R_{i,j}|\theta_{i},\theta_{j})P(\Theta )$$

Using *j* as the primary index and expressing the range of *i* in terms of *j*, we rewrite Formula (3):

(4) $$=\arg\underset{\Theta }{\max}\left\{\prod_{2\leq j\leq n}\left(\prod_{i=\max(1,j-k+1)}^{j}P(R_{i,j}|\theta_{i},\theta_{j})\right)P(\Theta )\right\}$$

We then divide the entire range of j=1,…,n into j≤k and j=k+1,…,n so that we can design an HMM algorithm for solving this optimization problem:

(5) $$\begin{array}{c} =\arg\underset{\Theta }{\max}\{\left(\prod_{1\leq i<j\leq k}P(R_{i,j}|\theta_{i},\theta_{j})\right)\\ \times \prod_{j=k+1}^{n}\left(\prod_{i=j-k+1}^{j-1}P(R_{i,j}|\theta_{i},\theta_{j})\right)P(\Theta )\} \end{array}$$

In the above formula, note that $P(R_{i,j}|\theta_{i},\theta_{j})$ is independent of contig orientations other than θ_*i*_ and θ_*j*_, and is also independent of other sets of contacts $R_{i\prime ,j\prime }$ where i≠i′ or j≠j′. Finally, we have:

argmaxΘP(R,Θ)=argmaxΘ{P(⊔1≤i<j≤kRi,j|(θl)l=1k)×∏j=k+1nP(⊔i=j−k−1j−1Ri,j|(θl)l=j−k+1j)P(Θ)},

where ${\left(\theta_{l}\right)}_{l=1}^{k}$ represents set $\left\{\theta_{1},\ldots ,\theta_{k}\right\}$. We can solve the above optimization problem using HMM such that the hidden states are sets of orientations:

$$S_{t}={\left(\theta_{l}\right)}_{l=t}^{t+k-1}\;\;\;\text{for}\;\;t=1,\ldots ,n-k+1,$$

The observations are sets of contacts:

(6) $$O_{t}=\{\begin{array}{cc} \underset{1\leq i<j\leq k}{⊔}R_{i,j} & \;\text{if}\;\;t=1\\ \underset{t\leq i\leq t+k-2}{⊔}R_{i,t+k-1} & \;\text{if}\;\;1<t\leq n-k+1, \end{array}$$

The emission probability $P(O_{t}|S_{t})$ is defined as:

$$P(O_{t}|S_{t})=\{\begin{array}{cc} P\left(\underset{1\leq i<j\leq k}{⊔}R_{i,j}|{\left(\theta_{l}\right)}_{l=1}^{k}\right) & \;\text{if}\;\;t=1\\ P\left(\underset{t\leq i\leq t+k-2}{⊔}R_{i,t+k-1}|{\left(\theta_{l}\right)}_{l=t}^{t+k-1}\right) & \;\text{if}\;\;1<t\leq n-k+1, \end{array}$$

which can be calculated using Formula (1), and the transition probability is:

$$\begin{array}{c} P(S_{t+1}|S_{t})\\ =\{\begin{array}{cc} \frac{1}{2} & \text{if}\;\text{orientations}\;\theta_{t+1},\ldots ,\theta_{t+k-1}\;\text{are}\;\text{consistent}\;\text{in}\;S_{t}\text{and}\;S_{t+1}\\ 0 & \text{otherwise} \end{array} \end{array}$$

**Fig. 3.**
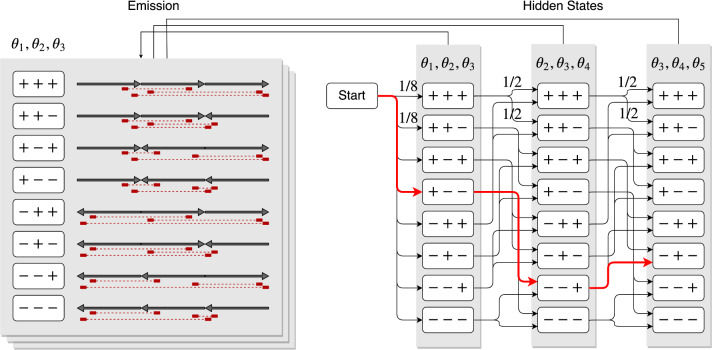
A sketch of the hidden Markov model proposed in this study, for the case where *k *=* *3 and *n *=* *5. The hidden states and all transitions are shown on the right, and the corresponding observations are shown on the left. For example, $\left(\theta_{1},\theta_{2},\theta_{3})=(+,-,-\right)$ is chosen because its corresponding observation is the most probable with the minimum sum of distances between pairs of contacts; therefore, its emission probability is the highest; that is, the product of probabilities of Hi-C contact pairs is the highest among the eight contig orientation patterns. For the other two hidden states, suppose that $\left(\theta_{2},\theta_{3},\theta_{4})=(-,-,+\right)$ and $\left(\theta_{3},\theta_{4},\theta_{5})=(-,+,-\right)$ are selected. The most likely path in this model, which is shown in red, represents the most probable orientations of the contigs (+,−,−,+,−)

Figure 3 illustrates an example of this HMM in the case of *k *=* *3 and *n *=* *5. The most likely path on the above HMM, which can be found efficiently using the Viterbi algorithm, gives the most probable set of contig orientations, Θ.

We have so far assumed that contigs are of the same size for better readability of the proposed algorithm. More precisely, for determining the orientation of the *i*th contig, we use contact information from the *j*th contig within *k* contigs (|i−j|<k) for a given constant *k*. In general, however, contigs differ in size. For handling regions wherein shorter contigs (e.g. <100 kb in length,) are enriched, contacts from more distal contigs would be informative to determine the contig orientations. In contrast, to process adjacent large contigs (e.g. >1 Mb in size), contacts between the two contigs would be sufficient. Thus, we must change the neighborhood of the focal *i*th contig adaptively, depending on the sizes of the neighboring contigs. To achieve this, hereafter, we will consider contacts between the *i*th and *j*th contigs within *K* distance $\sum_{i<s<j}L_{s}<K$. Recall that *K* is the threshold parameter for pairs of distal contigs in the Hi-C contact probability distribution *p*(*d*); when all contacts between the *i*th and *j*th contigs are at distance *K* or more ($\sum_{i<s<j}L_{s}\geq K$), they have the same probability regardless of their orientations.

Thus, the problem can be rewritten by setting $k_{0}=\min\{j|\sum_{1<s<j}L_{s}\geq K\}$ and $k(j)=\min\{j-i+1|\sum_{i<s<j}L_{s}\geq K\}$:

argmaxΘP(R,Θ)=argmaxΘ{P(⊔1≤i<j≤k0Ri,j|(θl)l=1k0)∏j=k0+1nP(⊔i=j−k(j)+1j−1Ri,j|(θl)l=j−k(j)+1j)P(Θ)},

which can be treated as an HMM with corresponding states and emission/transition probabilities. When determining orientations in regions with short contigs, the above modification allows us to use a sufficient number of contig orientations to correct errors. This idea is novel, and thus not described in the previous dynamic programing algorithm (Putnam *et al.*, 2016); moreover, it is useful for handling short contigs.

## 3 Experimental results

### 3.1 Datasets and error metrics

To demonstrate how misoriented contigs in 3D-DNA scaffolds are corrected in the HiC-Hiker package, we used human and worm genome datasets for benchmarking, since Hi-C and Illumina short-read data from these species are abundant, and their reference genomes are nearly complete.

We used the human genome dataset provided in Dudchenko *et al.* (2018). The first 300 million read pairs from the Illumina dataset for NA12878 of the Genome in a Bottle Consortium were assembled using the w2rap-contigger assembler (Clavijo *et al.*, 2017a, b). The first 100 million read pairs from the HIC001 *in situ* Hi-C library (Rao *et al.*, 2014) were mapped onto the contigs using the Juicer platform, with approximately 7× coverage. We assembled Illumina short reads (DRR008443, DRR008444) to create a worm genome dataset using the SPAdes v. 3.13.0 assembler (Bankevich *et al.*, 2012). We also used w2rap contigger; however, it output excessive chimeric contigs, which were not used in further analyses. The first 10, 20 and 40 million read pairs (7×, 14× and 28× coverage, respectively) from the Hi-C library (accession nos. SRR3105476 and SRR3105477; Gabdank *et al.*, 2016) were mapped onto the contigs using Juicer.

We then ran the entire 3D-DNA pipeline (version 180922) using these data with the default parameter settings, with the exception of minimum contig length, which was set to 5, 15 and 50 kb for the worm genome dataset to determine how its variation affected the accuracy of the results. We also ran two alternative scaffolding algorithms, SALSA2 (Ghurye *et al.*, 2019) and ALLHiC v. 0.9.13 (Zhang *et al.*, 2018, 2019), using the worm dataset. Hi-C reads were mapped using the BWA-MEM alignment package with the recommended settings of the scaffolding algorithms (Li and Durbin, 2010). We compared SALSA2 orientation corrections using assembly graphs and tested the results against NA12878 unitigs assembled from Oxford Nanopore reads using the Canu assembler (Koren *et al.*, 2017) and scaffolds generated by SALSA2 with and without the assembly graph shared by the creators of SALSA2 (Ghurye *et al.*, 2019).

We manually corrected large-scale (>1 Mb) errors in the 3D-DNA human scaffolds using Juicebox Assembly Tools (JBAT), such that 95 fragmented scaffolds were joined into 23 scaffolds, each of which corresponded to an individual human chromosome. No errors in the worm scaffolds were sufficiently large to require manual inspection.

**Fig. 4.**
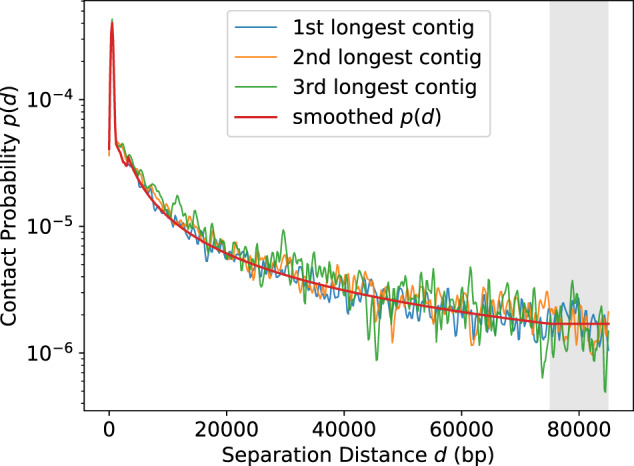
Comparison of contact probability distributions among the longest contig (blue), second longest contig (yellow) and the third longest contig (green). The smoothed distribution calculated from the longest contig is shown by the red line, which is actually used as *P*(*d*). This distribution appears to represent a good approximation of the top three distributions. *P*(*d*) was set to a constant probability when d> 75 kb, shown as a gray-colored region

Afterwards, we ran our software, HiC-Hiker, and inferred the probabilistic distribution *p*(*d*) based on the longest contig. To validate the feasibility of using the longest contig, we compared the distributions calculated from the three longest contigs in the human dataset and confirmed the consistency among the probability distributions (see Fig. 4). In Figure 4, the red line shows the fitted probability distribution, which was actually used as *p*(*d*). For the fitting, we used $p(d)=c_{1}d^{-c_{2}}$ as a base function; except for the probability of the short distance contacts (e.g. d< 3 kb), we used polynomial fitting to simulate the non-monotonically decreasing distribution actually observed. We set *K* to 75 kb, the third quantile of the histogram of Hi-C contact probability distribution. Note that the probability was unstable and noisy when *d* exceeded *K*.

After generating the refined scaffolds, we calculated the error rates. Given a scaffold with a series of contigs $A_{1},A_{2},A_{3},A_{4},B_{1},B_{2},B_{3}$, suppose that these contigs are aligned with the reference genome according to the following ordering and orientation:

$$A_{1},\bar{A_{2}},A_{3},A_{4},\bar{B_{3}},\bar{B_{2}},\bar{B_{1}}$$

where $\bar{X}$ represents a situation wherein the reverse complement of contig *X* is aligned. This example corresponds to the dot plots in Figure 1-(2). Our HiC-Hiker fixes *A*_2_, a misorientation of a single contig $\bar{A_{2}}$, by using the contacts from *A*_2_ to *A*_1_, *A*_3_ and *A*_4_. In contrast, however, $\bar{B_{3}},\bar{B_{2}},\bar{B_{1}}$ show a large erroneous inversion of $B_{1},B_{2},B_{3}$. HiC-Hiker is tailored to correct local errors, and is unlikely to handle this type of large-scale error; this necessitates a priori manual correction using the 3D-DNA GUI function.

With this example in mind, we here define global and local orientation errors. The orientation of a contig *X* is ‘globally erroneous’ if $\bar{X}$ is aligned with the reference. For example, in the running example, the orientations of $A_{2},B_{1},B_{2},B_{3}$ are globally erroneous. In contrast, the orientation is ‘locally erroneous’ if it is inconsistent with the orientations of its two neighboring contigs in the reference genome. According to this definition, only *A*_2_ is locally erroneous; $B_{1},B_{2},B_{3}$ are not. It would be reasonable to focus on correcting local errors if we assume that global orientation errors of large contig blocks (e.g. $B_{1},B_{2},B_{3}$) can mostly be corrected manually using JBAT. Thus, to demonstrate the reliability of contig orientations after refining the orientations of scaffolds with HiC-Hiker, we use the local error rate, that is, the ratio of contigs whose orientations are locally erroneous to all contigs.

Specifically, since the definitions of global and local errors differ, methods for detecting each error type must be designed individually. To calculate a *global orientation accuracy*, the researchers who introduced 3D-DNA randomly sampled 1000-nt regions from the scaffolds that are uniquely aligned onto the reference, and computed the ratio of the chunks oriented in agreement with the reference to the entire set of sampled regions (Dudchenko *et al.*, 2018). In contrast, to calculate a *local orientation accuracy*, we align contigs to the reference, and select triples of neighboring, uniquely mapped contigs such that their orderings does not change in the reference, and compute the ratio of the middle contigs whose orientations agree with those of their neighboring contigs to the entire set of sampled contigs, on both sides.

We also calculated the accuracy statistics suggested by Dudchenko *et al.* (2018), including anchoring accuracy (i.e. the percentage of chunks mapped onto the correct chromosome corresponding to each chromosome-length scaffold), global ordering accuracy (i.e. the percentage of randomly selected pairs of chunks mapped onto the correct chromosome in the same order as the reference sequences), local ordering accuracy (i.e. the percentage of adjacent pairs of chunks mapped onto the correct chromosome in the same order as the reference sequences) and global orientation accuracy (i.e. the percentage of chunks mapped onto the correct chromosome with the correct orientation). We calculated the average of each statistic over five different chunks.

As a reference sequence for the NA12878 human genome dataset, we adopted NA12878 inversions (Shao *et al.*, 2018) to the hg38 reference and modified the hg38 reference accordingly. As a reference sequence for the worm dataset, we used the latest complete VC2010 reference genome (Yoshimura *et al.*, 2019).

### 3.2 Comparison of HiC-Hiker with 3D-DNA

Local contig orientation errors were successfully corrected using our program. A comparison of dataset and scaffold accuracy metrics before and after running HiC-Hiker is shown in Table 1. At a minimum length threshold of 15 kb and 7× Hi-C coverage, which is the recommended configuration of the 3D-DNA tool suites, HiC-Hiker improved the accuracy of local ordering, global orientation and local orientation in the human and worm datasets. Scaffold accuracy was dependent on the threshold *K* value. A threshold of 7.5 kb was too short to improve scaffold accuracy, as demonstrated in the distribution plot; however, no significant difference in accuracy was observed between 75 and 200 kb. Therefore, 75 kb was selected as the optimal *K* threshold in terms of computation time and scaffold accuracy. The minimum length threshold affects the number of short contigs, which are difficult to order and orient, and are major targets of HiC-Hiker. Compared to lower (5 or 15 kb) thresholds, there are few short contigs that can be improved by examining in detail when with the higher threshold (50 kb), so HiC-Hiker offered only limited improvement. As the Hi-C coverage increased, the local accuracy of the intact 3D-DNA output improved. Orientation errors were successfully corrected even at 28× coverage using HiC-Hiker. The ALLHiC software generated chromosome-length scaffolds with accuracy values similar to those of 3D-DNA; ALLHiC was more accurate in terms of local layout but less accurate in terms of anchoring and global ordering. Even in this case, the local orientation of output scaffolds was slightly improved using the HiC-Hiker package. In the ALLHiC algorithm, the number of chromosomes is supplied beforehand, thus improving chromosome reconstruction, but limiting the applicability of ALLHiC to organisms with a known number of chromosomes. The SALSA2 algorithm is designed to handle long-read contigs to output highly accurate chromosome-length scaffolds. When short-read contigs were supplied, SALSA2 generated only fragmented scaffolds; thus, accuracy statistics could not be computed for chromosome-length scaffolds, and the HiC-Hiker algorithm could not be applied under these conditions. Because N50 contig lengths are sufficiently high, only a few short contigs required orientation correction; therefore, the improvement in accuracy under the HiC-Hiker package was nominal.

**Table 1.** A comparison of dataset and scaffold accuracy metrics before and after running HiC-Hiker

Species	Human (NA12878)		Worm (VC2010)									
Assembler	w2rap + Illumina	Canu + ONT	SPAdes + Illumina									
Contig N50 (kb)	68.6	4821.0	41.0									
Contig maximum length (kb)	934.6	34 607.7	283.5									
Scaffolding software	3D-DNA	SALSA2		3D-DNA							ALLHiC	SALSA2
		W/o graph	W/ graph									
Minimum length threshold	15k	—	—		15k		5k	50k	15k		15k	15k
Hi-C coverage	7×	7×	7×		7×		7×		14×	28×	7×	7×
Threshold K	75 kb	75 kb	75 kb	7.5 kb	75 kb	200 kb	75 kb		75 kb		75 kb	75 kb
Raw scaffolds >1 Mb before (after) manual correction												
No. of scaffolds	95 (23)	75	81		6		7	6	6	6	6	5
Total bases (Mb)	2391.4 (2392.0)	2624.8	2622.8		77.5		89.8	39.4	77.9	78.0	77.8	7.4
Maximum length (Mb)	127.7 (205.1)	273.4	188.9		16.0		18.7	8.8	16.1	16.2	16.2	1.8
Anchoring	99.75% (99.74%)	90.42%	96.76%		99.03%		95.89%	95.31%	98.89%	98.61%	98.24%	—
Ordering	98.39% (89.62%)	90.95%	93.20%		99.79%		99.37%	99.62%	99.82%	99.81%	98.93%	—
Local ordering	90.58% (89.97%)	90.24%	92.41%		85.71%		84.71%	84.25%	89.04%	91.20%	91.57%	—
Orientation	91.57% (90.95%)	90.31%	92.48%		86.31%		85.37%	84.82%	89.72%	91.95%	92.46%	—
Local orientation	95.72% (95.70%)	96.00%	96.34%		88.35%		86.07%	87.24%	89.83%	92.04%	93.01%	—
HiC-Hiker output												
Anchoring	99.74%	90.44%	96.75%	99.03%	99.00%	99.01%	95.17%	95.32%	98.90%	98.63%	98.23%	—
Ordering	89.64%	88.42%	94.74%	99.76%	99.80%	99.81%	99.26%	99.64%	99.82%	99.84%	98.98%	—
Local ordering	91.09%	90.42%	92.28%	81.06%	88.89%	89.11%	85.57%	84.65%	92.43%	93.93%	92.28%	—
Orientation	92.19%	90.49%	92.36%	81.51%	89.61%	89.85%	86.58%	85.20%	93.28%	94.78%	93.06%	—
Local orientation	98.25%	96.67%	96.73%	84.45%	93.75%	93.69%	92.06%	88.78%	95.83%	96.68%	93.80%	—
No. of contig flip												
True positives	1139	33	39	119	118	115	207	31	125	96	38	—
True negatives	35 157	1124	1122	1179	1323	1325	1962	317	1439	1507	1505	—
False positives	193	30	31	179	35	33	66	25	27	19	25	—
False negatives	453	34	30	60	61	64	122	19	41	36	77	—

**Fig. 5.**
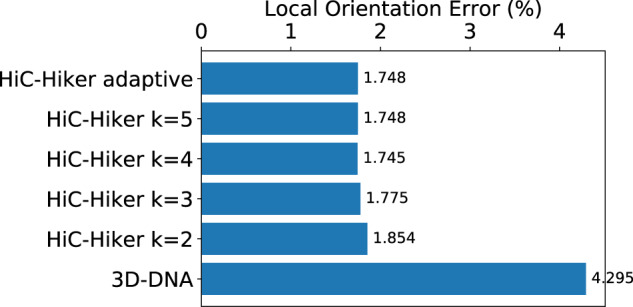
Local error rates of the human scaffolds generated by 3D-DNA and refined by HiC-Hiker. The error rate was remarkably reduced from 4.3% (3D-DNA) to 1.7% (HiC-Hiker)

Figure 5 shows the local error rates of scaffolds output by 3D-DNA and scaffolds refined by HiC-Hiker in human dataset, according to different numbers of hidden states, *k*. The local error rate in the scaffold generated by 3D-DNA was 4.3%, but was remarkably reduced to 1.7% after refinement via HiC-Hiker (adaptive). When we set the parameter *k* to 2, 3, 4 and 5, which represents how many contigs are considered in single hidden states, the local error rates were 1.85%, 1.78%, 1.75% and 1.75%, respectively. Thus, using contacts between more distal contigs slightly improves the accuracy of their orientations.

**Fig. 6.**
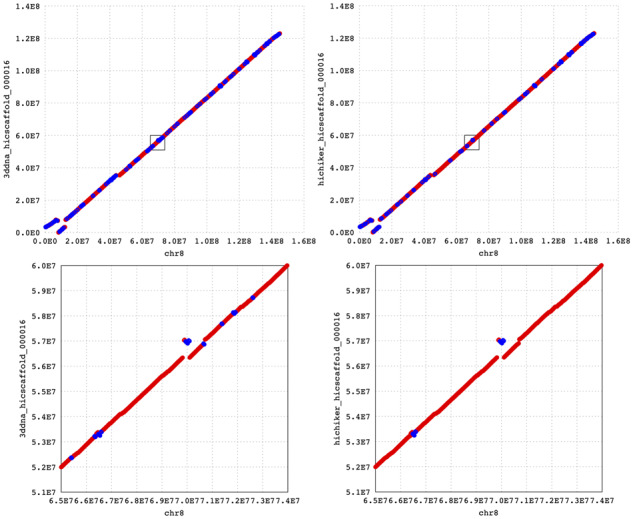
Four dot plots of scaffolds along human chromosome 8. The top row shows dot plots where the reference genome is on the x-axis and scaffolds output by 3D-DNA (left) and HiC-Hiker (right) are on the y-axis. The red-colored dots indicate correct orientations of contigs (forward alignment with the reference) while the blue-colored dots show erroneous orientations (reverse-complement alignments). The dot plots show that the reference is mostly covered by contigs. The total length of contigs in the scaffolds is 123 011 808 bp, which is close to 145 138 636 bp, i.e. the length of chromosome 8 in hg38. In the left bottom portions of both of the upper dot plots, we see large reverse-complement alignments of the reference and scaffolds. In the lower dot plots, we enlarged parts of the upper two plots to show the reference genomic region, which ranges from 65 to 74 Mb. The six orientation errors of short contigs shown in the lower left plot shown as blue dots are corrected in the HiC-Hiker scaffold shown in the lower right plot

Figure 6 shows dot plots of scaffolds along human chromosome 8, where the dot plots were generated using MUMmer4 (Marçais *et al.*, 2018). The left and right columns show 3D-DNA and HiC-Hiker scaffolds, respectively, in comparison with the reference genome. Blue dots show misoriented contigs, and the decrease in the number of the blue dots demonstrates the usefulness of refining orientations with HiC-Hiker.

**Fig. 7.**
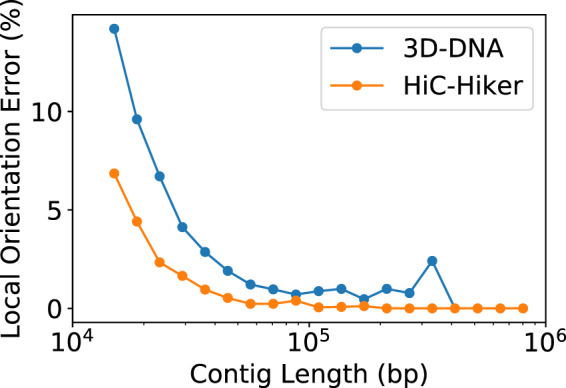
Plot of local error rates of contigs according to their lengths. Overall, shorter contigs are difficult to orient due to insufficient Hi-C contacts, but this plot indicates that HiC-Hiker outperformed 3D-DNA in repairing the orientations not only of shorter contigs, but also of longer contigs

Shorter contigs are likely to be more difficult to orient correctly than longer contigs, because fewer contacts are available on shorter contigs. To confirm this tendency, Figure 7 presents another plot showing the local error rates of contigs according to their lengths. In this analysis, contigs smaller than 15 kb in length were not considered because 3D-DNA was set to ignore them. This figure illustrates that it tends to be difficult to determine the orientations of shorter contigs, and HiC-Hiker can correct more local orientation errors in both short and long contigs than 3D-DNA.

For the human dataset, it took 37 min to run the whole pipeline on our computer, but the loading of contigs and Hi-C read mapping data spent most of the time. In particular, the HMM optimization process took 2, 4, 13 and 39 s when we set *k* to 2, 3, 4 and 5, respectively. Of note, the calculation time of the optimization depends on *k* exponentially. When we use adaptive mode, it took 20 s.

**Fig. 8.**
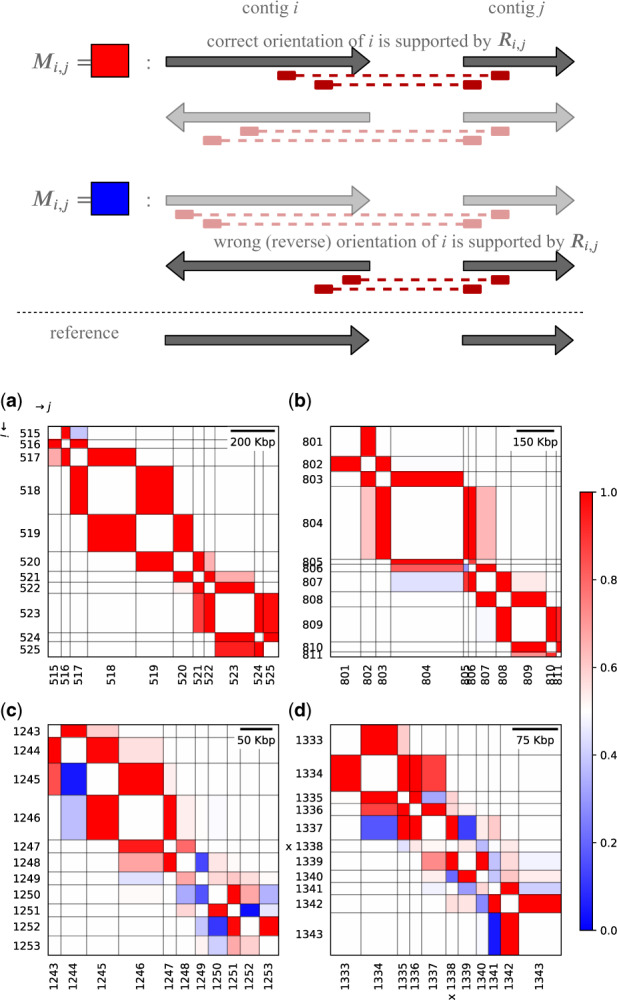
Relative frequency matrices $M_{i,j}$ for four typical cases. The identifiers of individual contigs, *i* and *j*, are shown beside the vertical and horizontal axes, respectively. The red-blue color of each cell shows the relative probability of its orientation. The schematic figure at the top illustrates that a red-colored cell ($M_{i,j}=1$) means that contacts between the *i*th and *j*th contigs determine the correct orientation of the *i*th contig, while a blue-colored cell ($M_{i,j}=0$) shows that contacts between the two contigs disagree with the correct orientation of the *i*th contig but support the wrong orientation erroneously. The top left matrix (**a**) shows the ideal situation, in which each contig is long enough to have sufficient contacts with its neighbors to allow its orientation to be determined before using HiC-Hiker. Since almost all of the contigs in this region are longer than the threshold of K= 75 kb in the probabilistic model, their orientations could be determined based only on their adjacent contigs; contacts with distant contigs were not required. In the top right (**b**), the row labeled with 806 shows a case where the orientation of contig 806 cannot be correctly determined according to its contacts with contig 805, which is depicted by the blue-colored cell of 805 and 806. HiC-Hiker corrected the misoriented contig 806 by considering its contacts with long contigs 804 and 807. In the bottom left (**c**), the row labeled with 1248 illustrates a situation where the misoriented contig 1248 is difficult to fix if its neighboring contigs 1249 is taken into account, but can be fixed using 1247 and 1250, all of which are small contigs (<50 kb). In the bottom right (**d**), the row labeled with 1338 shows a case when HiC-Hiker fails to correct the misoriented contig 1338 labeled with ‘x’, which has short contigs on its right side. The relative probability of contig 1338 being close to 1/2 (in the row 1338) indicates insufficient information regarding the orientation of 1338; it is difficult to determine its orientation based on neighboring contigs

To examine why this algorithm can improve the accuracy of contig orientations, we here define the relative orientation probability matrix *M*, such that

$$M_{i,j}=\frac{\sum_{\theta_{j}\in \{+,-\}}P(R_{i,j}|\theta_{i}=\hat{\theta_{i}},\theta_{j})}{\sum_{\theta_{i}\in \{+,-\}}\sum_{\theta_{j}\in \{+,-\}}P(R_{i,j}|\theta_{i},\theta_{j})},$$

where $\hat{\theta_{i}}$ represents the correct orientation of the *i*th contig. $M_{i,j}$ indicates the likelihood that the orientation of the *i*th contig is correct, which in turn informs our confidence in the orientation of the *i*th contig, given Hi-C contacts between the *i*th and *j*th contigs. Notably, *M* is asymmetric. Figure 8 shows typical examples of the matrix. In this plot, to represent the dependency of orientation accuracy on contig size, the horizontal and vertical lengths of each cell $M_{i,j}$ are proportional to the lengths of the *i*th and *j*th contigs, respectively.

Our method has two main advantages with respect to orienting contigs. First, it estimates the proximity of two contigs more precisely according to the probability of contact, whereas previous studies simply use absolute values such as a separation distance or a contact count. Second, since the HMM algorithm is able to find the global optimum, it is proven to be less prone to errors even in the presence of local and abnormal contacts than the previous iterative contig-joining algorithm. These advantages of our proposed method can be clearly seen in the relative orientation probability matrix plot shown in Figure 8. The top left matrix (a) presents a case where the contact information of adjacent contigs is sufficient to determine the orientations of contigs. On the other hand, the top right and bottom left matrices (b, c) show cases where the probabilistic metric is needed. We require proximity information from more distal contigs to obtain correct orientations.

## 4 Discussion

In this article, assuming that most large global errors are corrected by using 3D-DNA, we demonstrated that HiC-Hiker can further reduce the local orientation error rate from 4.3% to 1.7% in real human datasets, and from 11.7% to 6.3% in worm datasets.

In general, greater coverage of Hi-C contacts may make it possible to estimate orientations more accurately using a larger number of Hi-C contacts between contigs, although generating more contacts is more expensive. Instead, we examined a cost-efficient approach using a Hi-C dataset of shallow 7× coverage, and demonstrated that our proposed method was effective in refining scaffolds even if only a shallow coverage Hi-C dataset is available. In fact, the DNA-ZOO project recommends shallow 7× coverage Hi-C datasets to create chromosome-length genome assemblies with 3D-DNA at low cost (Dudchenko *et al.*, 2018). Indeed, when applied to short-read contig datasets with recommended parameters (i.e. 15 kb minimum length threshold and >7× Hi-C coverage; columns 1, 5, 9 and 10 in Table 1), HiC-Hiker halved the local orientation error rate with a sufficient average recall (71%) and precision (82%). Therefore, our software program HiC-Hiker can be used as a complement to the powerful 3D-DNA program.

We assumed no gaps between adjacent contigs due to the difficulty in reliably estimating the gap distances based on Hi-C contacts. However, this assumption is unrealistic and should be revised in the future, because gaps are often filled with long repetitive elements and assemblers are unlikely to extend contigs at repetitive regions to avoid the ambiguity associated with contig extension. Despite the assumption, however, the experimental results demonstrated that our algorithm can refine scaffolds, thus showing the practical feasibility of the algorithm. Nevertheless, future work should attempt to eliminate this assumption by making use of probabilistic estimation data or other information, such as assembly graphs.

Also, in the probabilistic model *p*(*d*), we focused only on the most dominant term, i.e. the separation distances of Hi-C contact pairs, and did not consider the effects of other factors such as high-GC content regions, low sequence mappability in repetitive regions or inherent three-dimensional structures. Although benchmarking showed that the model was effective when not taking the other factors into consideration, ignoring these factors may lead to unwanted biases due to non-uniform coverage of Hi-C contacts; moreover, considering them may further reduce orientation error rates. Indeed, models aimed at identifying significant 3D structures in a chromosome consider the effect of restriction enzyme sites, GC content and mappability (Carty *et al.*, 2017). Future work should modify our model to consider these factors.

Finally, it is useful to take a probabilistic approach to refine contig ordering and automatically correct large inversion errors. A probability of Hi-C contact seems to be a more precise and theoretic metric than a separation distance or a contact count, which could lead to major improvements in scaffolding based on Hi-C data. Additionally, if we use probabilistic models to calculate the optimal layout of contigs, the probability, or reliability of the layout and scaffolds can be calculated. This reliability information of the generated assembly can be useful when it is used in subsequent analyses.
